# Aerobic Fitness and Neurocognitive Function Scores in Young Faroese Adults and Potential Modification by Prenatal Methylmercury Exposure

**DOI:** 10.1289/EHP274

**Published:** 2016-09-09

**Authors:** Youssef Oulhote, Frodi Debes, Sonja Vestergaard, Pal Weihe, Philippe Grandjean

**Affiliations:** 1 Department of Environmental Health, Harvard T.H. Chan School of Public Health, Boston, Massachusetts, USA; 2 Department of Occupational Medicine and Public Health, Faroese Hospital System, Torshavn, Faroe Islands; 3 Institute of Public Health, University of Southern Denmark, Odense, Denmark

## Abstract

**Background:**

Exposure to methylmercury was shown to decrease neural stem cell populations, whereas aerobic fitness has beneficial effects on the adult brain that relies on improved neurogenesis in the hippocampus.

**Objectives:**

We examined the association between aerobic fitness and neurocognitive outcomes at young adult age, along with the potential moderating effect of prenatal exposure to methylmercury.

**Methods:**

At age 22 years, 262 members of a Faroese birth cohort, established in 1986–1987, underwent a graded exercise test of aerobic fitness to measure maximal oxygen uptake (VO_2Max_). Their prenatal methylmercury exposure had been assessed from the mercury concentration in cord blood. We estimated cross-sectional associations between VO_2Max_ and multiple measures of neurocognitive function. In addition, we compared groups with low and high prenatal methylmercury exposure.

**Results:**

A 1 standard deviation (SD) increase in VO_2Max_ was associated with better scores on short-term memory and cognitive processing speed by 0.21 SD (95% CI: –0.04, 0.46) and 0.28 SD (95% CI: 0.02, 0.54), respectively. In the group with lower prenatal methylmercury exposure, a 1 SD increase in VO_2Max_ was associated with increased scores on cognitive processing speed by 0.45 SD (95% CI: 0.08, 0.81) and with a slightly lesser benefit in short-term memory. No such association was observed in the group with high prenatal methylmercury exposure.

**Conclusions:**

Higher aerobic capacity was associated with better performance in short-term memory and processing speed. However, prenatal methylmercury exposure seemed to attenuate these positive associations.

**Citation:**

Oulhote Y, Debes F, Vestergaard S, Weihe P, Grandjean P. 2017. Aerobic fitness and neurocognitive function scores in young Faroese adults and potential modification by prenatal methylmercury exposure. Environ Health Perspect 125:677–683; http://dx.doi.org/10.1289/EHP274

## Background

Adult neurogenesis occurring in the dentate gyrus of the hippocampus is a dynamic process regulated by both intrinsic and extrinsic factors (Aimone et al. 2014). Neurogenesis can be affected in both positive and negative ways by several factors, such as physical activity, aging, stress, and diet. Recent evidence from longitudinal and randomized studies suggests that aerobic fitness is positively associated with improvements in cognitive functions in the elderly (Colcombe and Kramer 2003; Hindin and Zelinski 2012; Smith et al. 2010) along with increases in volumes of the hippocampus (Erickson et al. 2011) and the prefrontal cortex (Colcombe et al. 2006). These beneficial effects appear to occur among all age groups including children (Chaddock-Heyman et al. 2014; Desai et al. 2015; Sardinha et al. 2014), adolescents (Herting and Nagel 2012), and young adults (Åberg et al. 2009; Hillman et al. 2005).

In contrast, methylmercury is a well-known neurotoxicant, where the prenatal period is a critical window for toxicity to the developing brain (Grandjean and Landrigan 2014; Karagas et al. 2012). Experimental studies have demonstrated a detrimental effect of early exposure to low concentrations of methylmercury on neural cell survival and proliferation, neural stem cell populations, and hippocampal size (Falluel-Morel et al. 2007; Gundacker et al. 2012; Sokolowski et al. 2013; Tamm et al. 2006). However, it is not known how these effects observed early in life might affect neurogenesis processes in adulthood, and no previous study has investigated the potential moderating effect of prenatal exposure to neurotoxicants, such as methylmercury, on the positive relationship between aerobic fitness and cognitive functions.

In this study of young adult members of a Faroese birth cohort, we hypothesized that prenatal exposure to methylmercury might adversely affect adult neurogenesis, thus attenuating the potential beneficial effect of aerobic fitness on cognitive functions. Because the effects of aerobic fitness might be specific to some cognitive domains (Smith et al. 2013), we assessed the relationship between aerobic fitness and multiple neurocognitive outcomes, and whether these associations were modified by prenatal exposure to methylmercury from maternal seafood diets.

## Methods

### Participants

A Faroese birth cohort study (*n* = 1,022) was established in 1986–1987 at the three hospitals in the Faroe Islands (Grandjean et al. 1992), and consenting members were invited to a follow-up examination at age 22 years. A subsample of 262 cohort members underwent fitness testing during a limited period, thus believed to be at random because it depended only on the examination schedule. All subjects responded to a questionnaire on past medical history, current health status, and lifestyle habits [see “Assessment of Participants’ physical activity (Questionnaire)” in the Supplemental Material]. The ethical review committee covering the Faroe Islands as well as the U.S. institutional review board approved the study protocol, and all participants provided written, informed consent.

### Aerobic Fitness Measurement

Participants underwent a progressive test performed on a mechanically braked cycle-ergometer (Monark AB, Vansbro, Sweden). They were asked to cycle for 5 min at a 75 Watt (W) load for females and 100W load for males, maintaining 60–70 rounds per minute throughout the test. After the first 5 min, loads were increased by 35W every 2 min until the participant was exhausted. Heart rate was monitored and an optimal test was defined as attained if the heart rate was ≥ 185 beats per minute and/or if the test leader evaluated the participant had reached exhaustion when the test was stopped by the participant. All participants received verbal encouragement from the test leader throughout the test. We estimated the maximum oxygen uptake (VO_2Max)_ from a maximal power output (MPO) as described by Andersen (1995). MPO was calculated as the Watts in the last completed workload, plus the increment in Watts of the last step divided by 120 sec, and multiplied by the number of seconds of the last step. Maximal oxygen uptake (VO_2Max_) in L/min was estimated using the formula: VO_2Max_ (L/min) = 0.16 + (0.0117 × MPO), and then divided by weight and expressed in mL/kg/min.

### Prenatal Methylmercury Exposure

Prenatal exposure to methylmercury was determined from mercury analysis of umbilical cord blood samples collected at birth (CB-Hg in μg/L). Briefly, blood samples from the umbilical cord were taken in 10-mL Abbott syringes equipped with Teflon-lined pistons, and kept deep-frozen during transport and storage until analysis (Grandjean et al. 1992). Mercury concentrations were determined using a UV-absorptiometer (Mercury Monitor 1235). The quality was secured by inclusion of quality controls and standard reference material samples from the National Institute of Standards and Technology (Gaithersburg, MD, USA) as previously described by Grandjean et al. (1992).

### Neurocognitive Functions

A battery of neuropsychological tests was administered to ascertain a broad range of cognitive and learning abilities (Debes et al. 2016). We used several subtests from well-established clinical test batteries: the Wechsler Intelligence Scale for Children revised (WISC-R) (Wechsler 1974), the Wechsler Adult Intelligence Scale revised (WAIS-R) (Wechsler 1981), the Wechsler Memory Scale third edition (Wechsler 1997), the Woodcock–Johnson III test of cognitive abilities (Woodcock et al. 2001), the Boston Naming Test (BNT) (Kaplan et al. 1983), and the California Verbal Learning Test (CVLT) (Delis et al. 1987). The neurocognitive assessment took place at the same day, and just before the aerobic fitness testing. Detailed information about the tests administered has been recently published (Debes et al. 2016) and can also be found in “Neuropsychological tests (From Debes et al. 2015)” in the Supplemental Material. A total of 878 cohort members had been previously examined by a less comprehensive battery at age 14 years (Debes et al. 2006).

### Covariates and Potential Confounders

We considered the following covariates as possible confounders in the association between VO_2Max_ and neurocognitive outcomes: sex, body mass index (BMI, continuous), smoking (never, occasional, regular), self-reported health status (excellent, very good, good, not particularly good), and self-reported physical activity (exercising hard or doing competitive sports, exercising some hours a week, walking or riding bicycle a few hours a week, inactive). As in previous studies (Debes et al. 2006, 2016), we also considered predictors of neurocognitive outcomes: maternal and paternal employment, maternal and paternal education/training, and mother’s intelligence (Raven score).

We selected the covariates retained in the final models using a combined approach. First, we used directed acyclic graphs (Greenland et al. 1999) to infer a minimal set of sufficient confounders. Second, plausible variables that changed the effect estimates by ≥ 10% were also retained.

### Statistical Analyses

We log-transformed (base 10) CB-Hg concentrations to address skewness. Depending on the covariate, we used *t*-tests or analysis of variance to examine univariate associations between VO_2Max_ and participants’ characteristics.

We explored associations between VO_2Max_ and neurocognitive test scores using linear regressions adjusting only for sex. Furthermore, we used structural equations modeling (SEM) to assess the relationship between VO_2Max_ and groups of neurocognitive outcomes. In this approach, we considered test scores from specific neurocognitive tasks as indicators of six underlying latent functions in accordance with the Cattell–Horn–Carroll three-stratum theory (Schneider and McGrew 2012): Short-term Memory, Verbal Comprehension and Knowledge, Psychomotor Speed, Visual Processing, Long-term Storage and Retrieval, and Cognitive Processing Speed (Debes et al. 2016). Additional methodological aspects of the construction of latent functions are described in “Methodology for structural equations modeling” in the Supplemental Material. In a second approach, we allowed the six neurocognitive functions to be indicators of a broader neurocognitive function (*g*). In the first model, all the six neurocognitive functions were regressed on VO_2Max_, whereas in the second model, only the global *g* factor was regressed on VO_2Max_, adjusting for the same set of covariates. In a final model, we allowed the latent functions to be indicators of two broader neurocognitive functions, where Cognitive Processing Speed and Short-term Memory reflected Cognitive Efficiency, whereas Verbal Comprehension and Knowledge, Visual Processing, Long-term Storage and Retrieval, and participant’s Raven scores indicated the construct of General Thinking abilities (Debes et al. 2016; Woodcock et al. 2001). These two broader constructs were then regressed on VO_2Max_, while again adjusting for the same covariates. Predictors of neurocognitive outcomes—maternal and paternal employment, maternal and paternal education/training, and mother’s Raven scores—were also aggregated in a single latent variable to reflect family background. Some participants had missing data for physical activity (*n* = 1), smoking status (*n* = 2), maternal and paternal employment (*n* = 17), maternal education/training (*n* = 18), paternal education/training (*n* = 17), maternal Raven score (*n* = 21), and single neurocognitive test scores [*n* between 1 and 5, except for block designs (*n* = 48)]. We imputed missing data using multiple imputations by chained equations. We specified an adequate number of iterations (*n* = 10) according to the proportion of missing information (White et al. 2011). Mean estimates and variances were computed from the 10 imputed data sets using Rubin’s rules (Rubin 1987).

In additional analyses, we explored the differences in the association between VO_2Max_ and neurocognitive outcomes among two groups of higher and lower prenatal exposure to methylmercury. We assessed the differences in associations using two cut-offs. In the first analysis, we split CB-Hg at the median (23.5 μg/L), whereas in the second analysis we split the CB-Hg at the 67th percentile (35 μg/L) and ran the analysis across two groups of low (< 35 μg/L; lower two tertiles) and high (≥ 35 μg/L; highest tertile) prenatal exposure to methylmercury. To account for residual confounding within mercury exposure strata, we included mercury concentrations (log_10_-transformed) as a covariate. Differences in the associations in the two exposure groups were tested by comparing the value of *d*/SE*_d_* to the standard normal distribution, where *d* is the difference between the two estimates, and 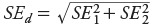 is the standard error of the difference (Altman and Bland 2003). An attempt to run SEM analyses across tertiles of CB-Hg failed to produce an acceptable model fit with lower numbers of observations.

In a sensitivity analysis, we attempted to adjust for potential selection bias due to incomplete participation or due to the exclusion of the individuals who did not perform optimally in the fitness test. We therefore applied stabilized inverse probability weights to all the models (Hernán et al. 2004) to conduct a weighted analysis. Because available SEM packages in R (version 3.2.3; R Project for Statistical Computing) do not allow for weighted analyses, we included these weights as sampling weights in a complex survey SEM analysis, while restricting clusters and strata to be the same for all individuals.

Because of the cross-sectional nature of the follow-up study and to ensure that potential significant associations between VO_2Max_ and neurocognitive functions were not due to reverse causality, we ran additional analyses to test whether participants with better neurocognitive functioning at 14 years of age had higher aerobic fitness at age 22 years. Because the neurocognitive assessment at age 14 years consisted of fewer subtests than at age 22 years (Debes et al. 2006), we considered test scores at 14 years of age as indicators of three latent functions based on the results from an exploratory factor analysis. We assessed the effect of each of these functions on VO_2Max_ in a SEM adjusting for sex, CB-Hg, and the family background latent variable. Finally, we considered the three factors as indicators of a broader neurocognitive function, and assessed its effect on VO_2Max_ at age 22 years, adjusting for the same covariates.

All tests were two-sided, significance was based on *z*-values from the Wald test, and analyses were conducted using the *Mice* (van Buuren and Groothuis-Oudshoorn 2011), *ipw* (Van der Wal and Geskus 2011), *lavaan* (Rosseel 2012), and *lavaan survey* (Oberski 2014) packages in R (version 3.2.3; R Project for Statistical Computing). Additional methodological aspects of SEMs are described in “Methodology for structural equations modeling” in the Supplemental Material.

## Results

Among the 262 participants who underwent the aerobic fitness test, 18 participants failed the test, and 47 performed suboptimally. Thus, only 197 performed optimally and had valid results of VO_2Max_. Women, nonsmokers, and physically active participants were significantly more likely to have an optimal aerobic fitness test. Participants who performed an optimal test were not different from those who did not in regard to CB-Hg, BMI, maternal and paternal employment and education/training, and maternal Raven score.

In univariate associations, VO_2Max_ was significantly higher among men, participants who had never smoked, individuals with normal BMI, and participants who reported being physically active and having an excellent state of health. No association with VO_2Max_ was observed regarding CB-Hg and maternal and paternal employment and education/training, nor with maternal Raven scores (Table 1).

**Table 1:** Levels of maximal oxygen uptake in relation to important covariates.

Participant characteristics	*n* (%)	VO_2Max_ [mL/kg/min] (mean ± SD)	*p*-Value
Sex			< 0.001
Women	77 (39)	31.2 ± 6.2
Men	120 (61)	37.6 ± 7.1
Smoking			0.001
Never	113 (58)	36.7 ± 7.2
Occasional	30 (15)	33.6 ± 6.5
Regular	52 (27)	32.4 ± 7.7
Missing	2	—
Prenatal mercury exposure			0.44
Low (< 23.5 μg/L)	99 (50)	34.7 ± 6.4
High (≥ 23.5 μg/L)	98 (50)	35.5 ± 8.3
BMI			< 0.001
< 25	111 (56)	37.7 ± 6.3
25–29	60 (31)	34.3 ± 6.4
> 29	26 (13)	25.8 ± 5.9
Self-reported physical activity			< 0.001
Practicing hard or competitive sports	46 (24)	40.5 ± 7.6
Doing sports some hours a week	48 (24)	36.1 ± 5.1
Walking or riding bicycle few hours a week	75 (38)	33.1 ± 6.3
Inactive	27 (14)	29.6 ± 7.4
Missing	1	—
Self-reported health status			< 0.001
Excellent	41 (21)	37.7 ± 6.5
Very good	78 (40)	37.2 ± 6.3
Good	71 (36)	31.7 ± 7.5
Not especially good	6 (3)	30.0 ± 10.1
Missing	1	—
Maternal education/training			0.88
No	71 (40)	35.4 ± 7.6
Yes	108 (60)	35.2 ± 7.6
Missing	18	—
Paternal education/training			0.88
No	43 (24)	35.4 ± 8.4
Yes	137 (76)	35.2 ± 7.3
Missing	17	—
Maternal employment			0.23
No	83 (46)	34.5 ± 7.7
Yes	97 (54)	35.9 ± 7.4
Missing	17	—
Paternal employment			0.70
No	28 (16)	34.7 ± 7.4
Yes	152 (84)	35.3 ± 7.6
Missing	17	—
Maternal Raven score			0.76
≤ 45	61 (35)	34.8 ± 8.0
46–51	62 (35)	35.8 ± 7.1
> 51	53 (30)	35.1 ± 7.3
Missing	21	—
Total	197 (100)	35.1 ± 7.4

All neurocognitive test scores exhibited appropriate variability, and were therefore adequate for subsequent analyses (see Table S1). Adjusting for sex, higher VO_2Max_ was significantly associated with higher scores of memory for words, CVLT scores on the learning trials 1–5, visual matching, and decision speed scores (see Table S2).

In a confirmatory factor analysis, neurocognitive test scores were adequate indicators of latent functions with significant standardized coefficients ranging between 0.25 and 0.93 (Table 2).

**Table 2:** Factor loadings and estimated correlation of measured test scores to the neurocognitive latent functions as previously defined (Debes et al. 2016).

Domain/neurocognitive test	Factor loading^*a*^	SE	*p*-Value	Standardized coefficient
Gsm
Numbers reversed	1	0	NA	0.85
Memory for words	0.35	0.08	< 0.001	0.59
Spatial span forward	0.25	0.08	0.002	0.28
Spatial span backward	0.24	0.08	< 0.001	0.34
Gc
Boston Naming Test, correct without cues	1	0	NA	0.78
Boston Naming Test, correct with cues	0.86	0.03	< 0.001	0.79
Synonyms	0.43	0.05	< 0.001	0.78
Antonyms	0.25	0.04	< 0.001	0.62
Verbal analogies	0.32	0.05	< 0.001	0.67
Gps
Finger tapping, dominant hand	1	0	NA	0.93
Finger tapping, non-dominant hand	0.86	0.11	< 0.001	0.70
Finger tapping, alternate hands	0.99	0.22	< 0.001	0.55
Gv
Block Design, WISC-R	1	0	NA	0.64
Block Design, WISC-R + 3 WAIS-R	1.66	0.20	< 0.001	0.66
Spatial Relations	0.74	0.17	< 0.001	0.70
Glr
CVLT, trial 1, number correct	1	0	NA	0.59
CVLT, learning trials 1–5, total number correct	6.92	0.72	< 0.001	0.83
CVLT, list B, number correct	0.72	0.13	< 0.001	0.38
CVLT, short delay, free recall, correct	1.72	0.28	< 0.001	0.91
CVLT, long delay, free recall, correct	1.78	0.30	< 0.001	0.92
CVLT, long delay, recognition, correct	0.45	0.11	< 0.001	0.42
Incidental Memory	0.83	0.24	0.001	0.25
Gs
Visual Matching	1	0	NA	0.83
Decision Speed	0.56	0.09	< 0.001	0.73
^***a***^For each neurocognitive function, the latent variable is constructed on the scale of the first component. Abbreviations: Gc, Verbal comprehension and knowledge; Glr, Long-term storage and retrieval; Gps, Psychomotor speed; Gs, Cognitive processing speed; Gsm, Short-term memory; Gv, Visual processing; NA, not applicable; SE, standard error.				

### Associations between Aerobic Fitness and Neurocognitive Functions

Final models included VO_2Max_, sex, physical activity, smoking status, BMI, family background, and prenatal methylmercury exposure (CB-Hg).

Although VO_2Max_ was positively correlated with all neurocognitive domains, the association was statistically significant only for cognitive processing speed (Table 3). We found that a 1-SD increase in VO_2Max_ was significantly associated with an increase in cognitive processing speed by 0.28 SD [95% confidence interval (CI): 0.02, 0.54; *z* = 2.14, *p* = 0.04]. In addition, a 1-SD increase in VO_2Max_ was associated with a marginally significant increase in short-term memory scores by 0.21 SD (95% CI: –0.04, 0.46; *z* = 1.66, *p* = 0.10).

**Table 3:** Adjusted associations between VO_2Max_ and neurocognitive functions.

Neurocognitive domain	B (95% CI)^*a*^	*p*-Value
Short-term memory	0.21 (–0.04, 0.46)	0.10
Verbal comprehension and knowledge	0.12 (–0.11, 0.34)	0.31
Psychomotor speed	0.03 (–0.18, 0.24)	0.78
Visual processing	0.07 (–0.24, 0.38)	0.64
Long-term storage and retrieval	0.16 (–0.06, 0.38)	0.16
Cognitive processing speed	0.28 (0.02, 0.54)**	0.04
Cognitive efficiency	0.32 (0.01, 0.62)**	0.04
General thinking abilities	0.16 (–0.10, 0.42)	0.23
General function (*g*)	0.24 (–0.02, 0.50)*	0.07
Models were adjusted for sex, physical activity, smoking status, BMI, family background, and prenatal methylmercury exposure. ^***a***^Change in the standard deviation of the neurocognitive function associated with a 1-SD increase in VO_2Max_. *p < 0.10. ** *p *< 0.05.		

In the SEM model assessing the association with general neurocognitive function (*g*), the six latent functions showed significant correlations to the *g*, with standardized coefficients between 0.61 and 0.76, except for motor function for which the standardized coefficient was weaker (0.25). A 1-SD increase in VO_2Max_ was associated with an increased neurocognitive *g* score of 0.24 SD (95% CI: –0.02, 0.50; *z* = 1.81, *p* = 0.07). In the final model using two broad neurocognitive functions, aerobic fitness was significantly associated with cognitive efficiency. Here, a 1-SD increase in VO_2Max_ was associated with a score increase of 0.32 SD (95% CI: 0.01, 0.62; *z* = 2.10, *p* = 0.04), although not in regard to general thinking abilities (Table 3).

### Effect Modification by Prenatal Methylmercury Exposure

In multiple group SEMs comparing the two groups with low (< 23.5 μg/L) and high (≥ 23.5 μg/L) prenatal methylmercury exposure, we found a significant positive association between aerobic fitness and cognitive processing speed in the group with lower prenatal methylmercury exposure, with a 1-SD increase in VO_2Max_ significantly associated with increased cognitive processing speed by 0.45 SD (95% CI: 0.08, 0.81; *z* = 2.45, *p* = 0.01). No clear association was observed for the other neurocognitive functions (Table 4). In the group with higher prenatal methylmercury exposure, we observed no association between VO_2Max_ and neurocognitive functions (Table 4). Although the association between VO_2Max_ and scores of cognitive processing speed was stronger among individuals with low prenatal methylmercury exposure compared to those with high exposure, the two groups did not differ significantly for any of the neurocognitive functions.

**Table 4:** Adjusted associations between VO_2Max_ and neurocognitive functions in regard to prenatal mercury exposure split at the median (23.5 μg/L).

Neurocognitive domain	B (95% CI)^*a*^		*p*_difference_
	Low prenatal exposure (< 23.5 μg/L)	High prenatal exposure (≥ 23.5 μg/L)	
Short-term memory	0.18 (–0.14, 0.50)	0.18 (–0.23, 0.60)	0.98
Verbal comprehension and knowledge	0.08 (–0.20, 0.35)	0.11 (–0.31, 0.52)	0.91
Psychomotor speed	0.12 (–0.16, 0.40)	–0.07 (–0.38, 0.25)	0.24
Visual processing	0.11 (–0.21, 0.43)	–0.02 (–0.53, 0.49)	0.60
Long-term storage and retrieval	0.16 (–0.13, 0.43)	0.19 (–0.15, 0.52)	0.90
Cognitive processing speed	0.45 (0.08, 0.81)**	0.16 (–0.24, 0.56)	0.20
Cognitive efficiency	0.43 (0.07, 0.80)**	0.24 (–0.20, 0.68)	0.53
General thinking abilities	0.15 (–0.17, 0.47)	0.15 (–0.22, 0.52)	0.97
General function (*g*)	0.27 (–0.05, 0.59)	0.18 (–0.18, 0.54)	0.69
Models were adjusted for sex, physical activity, smoking status, BMI, family background, and prenatal methylmercury exposure. ^***a***^Change in the standard deviation of the neurocognitive function associated with a 1-SD increase in VO_2Max_. ** *p *< 0.05.			

No significant association was found in the model assessing the association between VO_2Max_ and the general neurocognitive function (*g*), and the associations were similar in the two groups of exposure, although it was marginally significant in the lower exposure group (0.27 SD: 95% CI: –0.05, 0.59; *z* = 1.65, *p* = 0.10) (Table 4). However, we found a significant positive association between VO_2Max_ and cognitive efficiency in the group of low prenatal exposure, with a 1-SD increase in VO_2Max_ significantly associated with cognitive efficiency score increases by 0.43 SD (95% CI: 0.07, 0.80; *z* = 2.28, *p* = 0.02). Again, no clear association was found in the group of higher prenatal exposure (0.24 SD; 95% CI: –0.20, 0.68; *z* = 1.07, *p* = 0.28). No significant association was observed between VO_2Max_ and general thinking abilities in the two groups of lower and higher prenatal exposures (Table 4).

In the multiple group SEM analyses with a cut-off of prenatal methylmercury exposure of 35 μg/L (67% percentile), we observed the same trend as for the first cut-off at the median. Thus, we found a positive association between aerobic fitness and specific neurocognitive outcomes in the group with lower prenatal methylmercury exposure, with a 1-SD increase in VO_2Max_ associated with increased short-term memory and cognitive processing speed by 0.28 SD (95% CI: 0.00, 0.56; *z* = 1.96, *p* = 0.05) and 0.46 SD (95% CI: 0.19, 0.74; *z* = 3.22, *p* = 0.001). No significant association was observed for other neurocognitive functions, and none in the group with higher prenatal methylmercury exposure. However, in contrast to the analyses with a median cut-off, the associations differed significantly between the two groups of low and high prenatal exposure for cognitive processing speed (*p* = 0.007), though not for the other neurocognitive outcomes (Table 5).

**Table 5:** Adjusted associations between VO_2Max_ and neurocognitive functions in regard to prenatal mercury exposure split at the 67th percentile (35 μg/L).

Neurocognitive domain	B (95% CI)^*a*^		*p*_difference_
	Low prenatal exposure (< 35 μg/L)	High prenatal exposure (≥ 35 μg/L)	
Short-term memory	0.27 (0.00, 0.56)**	–0.08 (–0.55, 0.40)	0.21
Verbal comprehension and knowledge	0.01 (–0.27, 0.29)	0.14 (–0.25, 0.53)	0.57
Psychomotor speed	0.10 (–0.04, 0.23)	–0.15 (–0.44, 0.14)	0.12
Visual processing	0.25 (–0.06, 0.56)	–0.17 (–0.57, 0.22)	0.09
Long-term storage and retrieval	0.09 (–0.16, 0.34)	0.17 (–0.13, 0.47)	0.69
Cognitive processing speed	0.47 (0.19, 0.74)**	–0.12 (–0.44, 0.21)	0.007
Cognitive efficiency	0.49 (0.20, 0.79)**	–0.12 (–0.58, 0.33)	0.03
General thinking abilities	0.08 (–0.19, 0.35)	0.27 (–0.17, 0.71)	0.47
General function (*g*)	0.27 (–0.02, 0.55)*	0.13 (–0.21, 0.47)	0.54
Models were adjusted for sex, physical activity, smoking status, BMI, family background, and prenatal methylmercury exposure. ^***a***^Change in the standard deviation of the neurocognitive function associated with a 1-SD increase in VO_2Max_. *p < 0.10. **p < 0.05.			

When assessing the general neurocognitive function (*g*), the associations were similar in the two groups of exposure, although marginally better scores were seen in the lower exposure group (0.27 SD; 95% CI: –0.02, 0.55; *z* = 1.89, *p* = 0.06) (Table 5). Again, we observed the same trend when comparing results in the tertile groups of prenatal exposure in regard to the cognitive efficiency and general thinking abilities. Thus, we found a positive association in subjects with prenatal methylmercury exposure within the two lower tertiles, where a 1-SD increase in VO_2Max_ associated with increased cognitive efficiency (0.49 SD; 95% CI: 0.20, 0.79; *z* = 3.20, *p* = 0.001), but not general thinking abilities. The associations between VO_2Max_ and cognitive efficiency differed significantly between the highest tertile and the two lower tertiles of prenatal exposure (*p* = 0.03).

In the sensitivity analysis, estimates of associations adjusting for inverse probability weights led to similar conclusions as those drawn from main analyses, although the estimates were slightly attenuated (see Tables S3, S4, and S5).

### Associations between Neurocognitive Functions at Age 14 and Aerobic Fitness at Age 22 Years

The general neurocognitive score at age 14 was significantly correlated with the six latent cognitive functions at 22 years with correlations ranging from 0.24 (motor function) to 0.91 (short-term memory). Analyses assessing reverse causation showed no association between neurocognitive scores at 14 years of age and aerobic fitness at 22 years, with changes of 0.09, –0.04, and 0.06 SD in VO_2Max_ at 22 years for a 1 SD increase in age 14 neurocognitive factors 1, 2, and 3 (*p* = 0.25, 0.54, and 0.57, respectively). Likewise, a 1-SD increase in the general neurocognitive score at age 14 was not associated with VO_2Max_ at age 22 years (standardized β = 0.11 SD; 95% CI = –0.11, 0.34).

## Discussion

In the present study, aerobic fitness showed positive associations with scores of cognitive processing speed at age 22 years, but not in regard to other neurocognitive functions. The results also exhibited marginally significant associations for short-term memory and general neurocognitive function. Thus, a 1-SD increase in maximal oxygen uptake (mL/kg/min) was associated with increases in scores by 24%, 28%, and 21% of the SD for general neurocognitive function, cognitive processing speed and short-term memory, respectively. These results extend findings from previous studies on associations between aerobic fitness and neurocognitive functions (Chaddock-Heyman et al. 2014; Colcombe and Kramer 2003; Colcombe et al. 2006; Desai et al. 2015; Erickson and Kramer 2009; Erickson et al. 2011; Sardinha et al. 2014). Of particular interest, the positive association of aerobic fitness and cognitive processing speed and short-term memory appeared to be constrained to individuals with lower prenatal exposure to methylmercury. Furthermore, higher aerobic fitness in adulthood did not appear to be driven by better cognitive functioning during adolescence, thus arguing against reverse causation. Our study is the first to address the modifying effect of prenatal exposure to neurotoxicants on the potential positive relationship between aerobic fitness and cognitive functions.

Aerobic fitness was associated significantly only with cognitive efficiency scores indicated by cognitive processing speed and short-term memory, thereby suggesting that the influence of aerobic fitness on neurocognitive function might be specific for certain functional domains. Although test batteries vary widely between different studies, and domain-representation differs between tests, previous studies suggest that tests involving response speed may be the most sensitive to aerobic exercise and fitness (Etnier et al. 2006; Hillman et al. 2005; Smith et al. 2010, 2013). Spatial learning and memory may benefit more than verbal learning and delayed memory recall (Herting and Nagel 2012). These findings appear to be in accordance with the notion that exercise benefits memory encoding in the hippocampus, but not retrieval involving other brain regions (Rugg et al. 2002).

Several mechanisms might explain the link between aerobic fitness and neurocognitive functioning (Hötting and Röder 2013). Aerobic fitness has been shown to affect the hippocampus through increases in cerebral blood volume (Pereira et al. 2007) and densities of neuronal synapses (Kramer et al. 2002), in addition to the stimulation of neurogenesis (van Praag 2008; van Praag et al. 1999). After inhibition of neurogenesis, one study in mice showed no exercise-related enhancement in memory performance and spatial learning (Clark et al. 2008). Because neurogenesis must arise from a local neural stem cell population (Christian et al. 2014), the plasticity of the hippocampal neuronal circuit can be negatively affected by a decreased availability of neuronal stem cells.

The positive association between aerobic fitness and neurocognitive functioning appeared to be constrained mainly to individuals with lower prenatal methylmercury exposure, and no clear association was observed in individuals highly exposed to methylmercury in the womb. Because aerobic fitness is likely to exert its effect through neurogenesis, it is possible that prenatal exposure to methylmercury affected the cellular basis for this process. *In vitro* studies suggest that neural stem cells are exceedingly sensitive to adverse effects from mercury exposure (Gundacker et al. 2012; Tamm et al. 2006). In animal models, exposure to methylmercury during the perinatal period resulted in decreased neural stem cell populations (Sokolowski et al. 2013), inhibited hippocampal DNA synthesis, degradation of cyclin E, and reduced cyclin D1 and D3 during postnatal development in which neurogenesis is highly active in the hippocampus (Tyler and Allan 2013). It also induced apoptotic cell death (Falluel-Morel et al. 2007). All of these effects may result in subsequent deficits in hippocampal structure and function. Additionally, exposure to methylmercury has been reported to disturb the serotonergic system (Oudar et al. 1989), which plays a direct regulatory role in exercise-dependent hippocampal neurogenesis (Klempin et al. 2013).

Due to the limited number of cohort members included in this study, details on the exposure dependence of such effects cannot be estimated. Significant differences were seen only when comparing the highest exposures to the rest of the cohort. Within this cohort, about 90% of the subjects were prenatally exposed to methylmercury at levels exceeding the reference dose recommended by the U.S. Environmental Protection Agency (5.8 μg/L), but the study size did not allow meaningful comparisons between subjects with low background exposures to those with elevated exposures. Despite the nonsignificant effect modification reported when comparing participants above the median prenatal methylmercury exposure to those below the median, we speculate that stem cell–mediated toxicity may occur well below the median level of prenatal methylmercury exposure in the present study, given the overall tendencies and the well-documented and extreme sensitivity of neural stem cells to mercury exposure.

This study has some limitations. The cross-sectional design at the age 22 years follow-up examination does not allow causal inference, but we were able to exclude an impact of better cognitive outcomes at age 14 on aerobic capacity 8 years later. However, because fewer tests were administered at age 14 years, we were not able to directly compare the same neurocognitive functions, so reverse causation could not be completely ruled out. Exposure to methylmercury was assessed from the total mercury concentration in cord blood as a measure of the exposure during the last trimester of pregnancy. This parameter appears to be the best indicator of subsequent risk of mercury-associated cognitive deficits (Grandjean and Budtz-Jørgensen 2007; Grandjean et al. 2014), but may not necessarily reflect an effect on brain stem cells. Although the reduced number of subjects in the present study did not allow sufficient power to reveal significant associations between prenatal methylmercury exposure and neurocognitive functions, results from the full cohort have amply demonstrated persistent exposure-associated effects on cognition at age 22 years (Debes et al. 2016). Finally, the aerobic capacity of the participants in the present study must be interpreted to be low. The median VO_2max_ for males and females was 37.8 and 31.4 mL/min/kg, respectively, whereas the age- and sex-matched median VO_2Max_ in an American sample was 43.9 and 37.8 mL/min/kg for males and females, respectively (Pescatello and American College of Sports Medicine 2014). Although discrepancies in the methods applied may explain part of the difference (Shephard 1984), the lower aerobic capacity observed in this study is undoubtedly related to the high proportion of overweight and obese individuals in our study population (44%). Thus, at the fitness levels observed, the participants would likely have experienced only moderate cognitive benefits, thereby diminishing the sensitivity of the study.

We were able to adjust for several important confounders, including physical activity and predictors of neurocognitive function. Thus, education did not appear to affect our estimates. Nonetheless, possible residual or additional unmeasured confounding cannot be ruled out. Many other factors over the life course might be associated with both fitness and cognitive functions, including genetic factors, diet, and other socioeconomic parameters not captured in the present study. However, the homogeneity of the Faroese population in terms of sociodemographic, lifestyle, and genetic characteristics limits the potential influence of any unmeasured confounders. Another factor that may induce residual confounding is that methylmercury exposure mainly originates from seafood, which also contributes n-3 fatty acids that may enhance adult hippocampal neurogenesis and promote synaptic plasticity (Crupi et al. 2013), thus possibly mitigating deleterious effects of methylmercury due to negative confounding. Previous reports from this cohort showed that additional adjustment for maternal self-reported fish intake slightly strengthened the negative associations between methylmercury and cognitive functions (Debes et al. 2006; Budtz-Jørgensen et al. 2007). Unfortunately, the lack of precise measurements of nutrient supply (e.g., omega 3 fatty acids) precludes the assessment of whether such nutrients that could enhance hippocampal neurogenesis might also modify the association between aerobic fitness and cognitive functions.

The extended test battery allowed assessment of the association of aerobic fitness with a multitude of neurocognitive domains, as well as general neurocognitive function. The use of structural equation modeling resulted in an overall and more precise assessment of the outcomes compared to the classical approach where test scores are assessed individually. This approach takes into account measurement imprecision and also avoids inference errors arising from multiple comparisons. Still, the classification of the different tests is a challenge in regard to the complexity of the brain functionalities, and more than a single neurocognitive domain might be involved in specific performances.

## Conclusions

In conclusion, this study adds new insight to the mounting evidence suggesting positive associations of aerobic fitness with neurocognitive functions. Although the study size precluded a detailed assessment of this association at different levels of prenatal methylmercury exposure, elevated exposure levels appeared to attenuate the well-documented positive effect of aerobic fitness. The promotion of healthy lifestyles is becoming a recognized cost-effective public health intervention, and our findings suggest that further benefit in this regard may be achieved by early prevention of environmental exposures to neurotoxicants such as methylmercury.

## Supplemental Material

(259 KB) PDF
