# Supplementary material for: Aerobic Fitness and Neurocognitive Function Scores in Young Faroese Adults and Potential Modification by Prenatal Methylmercury Exposure
Source: Environ Health Perspect. 2016 Sep 9;125(4):677–83. doi: 10.1289/EHP274 (PMC5381980; doi:10.1289/EHP274)
Supplement: (259 KB) PDF [file EHP274.s001.acco.pdf]

Environ Health Perspect
DOI: 10.1289/EHP274

Note to readers with disabilities: *EHP* strives to ensure that all journal content is accessible to all readers. However, some figures and Supplemental Material published in *EHP* articles may not conform to 508 standards due to the complexity of the information being presented. If you need assistance accessing journal content, please contact ehponline@niehs.nih.gov. Our staff will work with you to assess and meet your accessibility needs within 3 working days.

Supplemental Material

Aerobic Fitness and Neurocognitive Function Scores in Young Faroese Adults and Potential Modification by Prenatal Methylmercury Exposure

Youssef Oulhote, Frodi Debes, Sonja Vestergaard, Pal Weihe, and Philippe Grandjean

Table of Contents

1. Neuropsychological tests (From Debes et al. 2015)

2. Methodology for structural equations modeling:

3. Assessment of Participants' physical activity (Questionnaire)

Table S1. Descriptive results of neurocognitive test scores.

Table S2. Associations between aerobic fitness and neurocognitive test scores.

Table S3. Adjusted associations between VO_{2max} and neurocognitive functions accounting for inverse probability weighting

Table S4. Adjusted associations between VO_{2max} and neurocognitive functions in regard to prenatal mercury exposure split at the median (23.5 µg/L), accounting for inverse probability weighting

Table S5. Adjusted associations between VO_{2max} and neurocognitive functions in regard to prenatal mercury exposure split at the 67th percentile (35 µg/L), accounting for inverse probability weighting

1. **Neuropsychological tests (From Debes et al. 2015)**

Woodcock-Johnson III (WJ III), Numbers Reversed**:** The test measures Short-Term Memory (Gsm) and Working memory (Schrank, 2001). The stimuli are Auditory (numbers) and require holding a span of numbers in immediate awareness while reversing the sequence by the cognitive processes of span of apprehension and recoding in working memory. Responses are Oral (numbers).

WJ III, Memory for words**:** The test measures Short-Term Memory by the narrow ability of auditory memory span (Schrank, 2001). Stimuli are auditory (words). The test requires repeating a list of unrelated words in a correct sequence by the formation of echoic memories and by the verbalizable span of echoic store. Responses are oral (words).

Wechsler Memory Scale III (WMS III), Spatial Span**:** The tests measures Short-Term Memory by the narrow ability of visual spatial span in a forward and in a backward condition (Schrank, 2001). The test is intended as a visual analogue to the Digit Span Test in the Wechsler scales. Stimuli are ten blue blocks randomly placed on a white form board. The examiner points out sequences of increasing length by touching a number blocks at a pace of one block per second. The subject has to reproduce a demonstrated sequence in the same order in the first condition, and in reverse order the second condition.

Boston Naming Test: The 60-item Boston Naming Test (BNT) (Kaplan, Goodglass, & Weintraub, 1983) is a visual confrontation naming test which measures the word retrieval or word finding performance of a subject. Stimuli are line drawings of a wide category of objects of increasing difficulty. Scores are obtained for number of correct items without cueing, and correct number of items after stimulus and phonemic cueing by the examiner.

WJ III, Synonyms, Antonyms, Verbal Analogies: Together these tests comprise Verbal Comprehension in WJ III and contribute to the CHC-factor Comprehension-Knowledge (Gc) by measuring the narrow abilities of Lexical Knowledge and Language Development (Schrank, 2001). Responses are oral (words). Nine items at adult level of difficulty from Picture Vocabulary, not overlapping with the Boston Naming Test, were also administered, but only included in scores of the Incidental Memory condition of the BNT.

WISC-R, Block Design (+ 3 last items from WAIS-R): To be consistent with the administration at age 14 years (Debes et al. 2006), where the three most difficult items from the adult version (WAIS-R) (Wechsler, 1981) were added to the children's version (WISC-R) (Wechsler, 1974),

the same combination of items was used at age 22 years. By an unfortunate error of administration, the three items from WAIS-R were not administered in the first part of the study, so that number of scores obtained for these items was reduced. The test measures Visual-Spatial Thinking (Gv) by narrow abilities for visuospatial perception, analysis, abstraction, synthesis and construction.

WJ III, Spatial Relations: The test measures Visual-Spatial Thinking by the narrow abilities of Visualization and Spatial relations (Schrank, 2001). The stimuli are visual (drawings). The test requires visual feature detection, manipulation of visual images in space and matching. Responses are oral (letters) or motoric (pointing).

California Verbal Learning Test (CVLT): The test measures learning, short-term and long-term retrieval as well as recognition (Glr) of a shopping list of sixteen items by cognitive component processes of maintaining information in immediate memory, learning by coding into long-term memory, recall by retrieval from long-term memory, semantic categorization, and matching of stimuli with newly stored content in long-term memory (Delis, Kramer, Kaplan, & Ober, 1994).

Incidental Memory: This added test condition measures long term memory and retrieval (Glr). After about 45 minutes the subjects were asked what pictures they incidentally could remember from the Boston Naming test and the Picture Vocabulary previously presented to the subject as described above.

WJ III, Visual matching: The test measures Processing Speed (Gs) by the narrow ability of Perceptual speed. Stimuli are visual (numbers) (Schrank, 2001). The task requires rapidly locating and circling identical numbers from a defined set of numbers by the process of speeded visual perception and Matching. The response is motoric (circling).

WJ III, Decision Speed: The test measures Processing Speed by the narrow ability of Semantic processing speed (Schrank, 2001). Stimuli are visual (pictures). The test requires Locating and circling two pictures most similar conceptually in a row by processes of object recognition and speeded symbolic/semantic comparisons. The response is Motoric (circling).

2. **Methodology for structural equations modeling:**

Neurocognitive latent functions at 22 years: Short-term memory was indicated by scores related to the subtests memory for words and numbers reversed from the WJ-III test, in addition to the scores of forward and backward spatial span from the WMS-III test. Verbal comprehension and

knowledge was indicated by the number of correct items with and without cues in the BNT, and scores in the synonyms, antonyms, and verbal analogies of the WJ-III. Psychomotor speed was indicated by scores in the finger-tapping tests of the NES2. Visual processing was indicated by scores of the block design items from the WISC-R and supplementing items from the WAIS-R, in addition to scores in spatial relations from the WJ-III. Long-term storage and retrieval was indicated by scores in the CVLT items, and scores of the incidental memory test based on remembering pictures from the BNT and WJ-III tests. Finally, cognitive processing speed was indicated by scores of visual matching and decision speed from the WJ-III test. We used a confirmatory factor analysis (CFA) to ensure the good discriminant validity between the six factors. Because the correlation between indicators of the same function might not be fully explained by the underlying latent variable, we allowed measurement errors of test scores indicating the same latent function, and we adjusted also for local dependence.

Neurocognitive latent functions at 14 years: The 1st factor reflected learning and memory, and was indicated by scores of four items from the CVLT test. The 2nd factor was indicated by three scores from the BNT, thus reflecting verbal knowledge and understanding. Finally, the 3rd factor was indicated by scores of the digit span and spatial span items, reflecting short-term memory.

Estimators and goodness of fit: We used the maximum likelihood estimator with robust standard errors to non-normality and non-independence of observations. We further assessed the models' goodness of fit using several indicators, i.e. the chi-square test, the root mean square error of approximation (RMSEA), the comparative fit index (CFI), and the standardized root mean square residual (SRMR). All models showed good to excellent fit to the data in regard to these indicators.

3. Assessment of Participants' physical activity (Questionnaire)

A. How much do you move while you are at work or at school on normal week days? (put one cross)

1 I do not work/do not attend school
2 I normally move a lot at work/at school
3 I normally move quite a lot at work/at school
4 I normally do not move much at work/at school
5 I normally do not move at work/at school

B. How much do you move to or from work, school or other place during a week? Walking, cycling etc. (put one cross)

1 I do not work/do not attend school
2 I normally do not move to or from work/school
3 In total, I move up to 1 hour a week to and from work/school
4 In total, I move 2 to 4 hours a week to and from work/school
5 In total, I move more than 4 hours a week to and from work/school

C. If you consider the last 12 months, which description fits you the best regarding, how much you move in your spare time? (put only one cross)

1 I do hard exercise or engage in competitive sport regularly and several times a week
2 I engage in sports but not on a competitive level a few hours a week
3 I walk, cycle or move in other ways a few hours a week
4 I move very little in my spare time

D. How many hours A DAY or A WEEK are you physically active?

Hours__________ a DAY or Hours___________ a WEEK

E. Do you have any hobbies? (such as sports, music, dance etc (put one cross))

1 No
2 Yes → If yes, please explain: ___________________________________

Table S1: Descriptive results of neurocognitive test scores.

Domain	Neurocognitive tests	n	Mean	SD	Min	Max
Gsm	Numbers reversed, WJ-III	195	15.89	3.47	9	28
	Memory for words, WJ-III	195	18.99	1.93	14	24
	Spatial span forward, WMS-III	195	9.15	1.71	5	14
	Spatial span backward, WMS-III	195	8.9	1.3	5	12
Gc	Boston Naming Test, correct without cues	196	48.9	5.06	29	58
	Boston Naming Test, correct with cues	196	51.79	4.28	35	59
	Synonyms, WJ-III	196	8.56	2.3	4	13
	Antonyms, WJ-III	196	12.9	1.89	5	17
	Verbal analogies, WJ-III	196	9.2	1.94	3	14
Gps	Finger tapping, Dominant Hand, NES2	192	172.74	21.36	136	255
	Finger tapping, Non Dominant Hand, NES2	192	165.54	24.88	122	255
	Finger tapping, Alternate Hands, NES2	192	237.97	35.53	135	396
Gv	Block design, WISC-R	195	57.45	6.12	17	62
	Block Design WISC-R + 3 WAIS-R	147	74.84	7.62	39	83
	Spatial relations, WJ-III	195	74.41	4.35	49	81
Glr	CVLT, Trial 1, Correct	196	5.81	1.72	2	12
	CVLT, Learning trials 1-5, Total Score Correct	196	49.7	9.03	20	72
	CVLT, List B, Correct	196	5.64	1.7	0	11
	CVLT, Short Delay, Free Recall, Correct	196	11.13	2.33	4	16
	CVLT, Long Delay, Free Recall, Correct	196	11.55	2.36	3	16
	CVLT, Long Delay, Recognition, Correct	195	14.99	1.15	9	16
	Incidental Memory	196	9.61	3.59	2	19
Gs	Visual matching, WJ-III	195	49.71	5.8	35	60
	Decision speed, WJ-III	195	36.31	3.8	25	40

SD: standard deviation; Gsm: short-term memory; Gc: comprehension and knowledge; Gps: psychomotor speed; Gv: visual processing; Glr: long-term storage and retrieval; Gs: cognitive processing speed; NES2: Neurobehavioral Evaluation System 2;WISC-R: Wechsler Intelligence Scale for Children revised; WAIS-R: Wechsler Adult Intelligence Scale revised; the WMS-III: Wechsler Memory Scale third edition; WJ-III: Woodcock-Johnson III test of cognitive abilities; BNT: Boston Naming Test; CVLT: California Verbal Learning Test (CVLT)

Table S2: Associations between aerobic fitness and neurocognitive test scores.

Domain	Neurocognitive tests	n	B[a]	95% CI	p-value
Gsm	Numbers reversed, WJ-III	195	0.25	0.04, 0.45	0.02
	Memory for words, WJ-III	195	0.12	-0.08, 0.33	0.24
	Spatial span forward, WMS-III	195	0.07	-0.13, 0.28	0.49
	Spatial span backward, WMS-III	195	0.05	-0.16, 0.26	0.62
Gc	Boston Naming Test, correct without cues	196	0.16	-0.05, 0.36	0.14
	Boston Naming Test, correct with cues	196	0.20	0.00, 0.41	0.06
	Synonyms, WJ-III	196	0.07	-0.14, 0.27	0.52
	Antonyms, WJ-III	196	0.10	-0.11, 0.30	0.36
	Verbal analogies, WJ-III	196	0.16	-0.05, 0.36	0.13
Gps	Finger tapping, Dominant Hand, NES2	192	0.02	-0.19, 0.22	0.88
	Finger tapping, Non Dominant Hand, NES2	192	-0.16	-0.36, 0.05	0.14
	Finger tapping, Alternate Hands, NES2	192	0.10	-0.10, 0.30	0.31
Gv	Block design, WISC-R	195	0.12	-0.08, 0.33	0.25
	Block Design WISC-R + 3 WAIS-R	147	0.04	-0.21, 0.30	0.73
	Spatial relations, WJ-III	195	0.08	-0.12, 0.29	0.44
Glr	CVLT, Trial 1, Correct	196	0.10	-0.10, 0.30	0.34
	CVLT, Learning trials 1-5, Total Score Correct	196	0.19	0.00, 0.38	0.05
	CVLT, List B, Correct	196	0.17	-0.03, 0.37	0.10
	CVLT, Short Delay, Free Recall, Correct	196	0.13	-0.07, 0.34	0.20
	CVLT, Long Delay, Free Recall, Correct	196	0.19	-0.01, 0.39	0.07
	CVLT, Long Delay, Recognition, Correct	195	0.00	-0.20, 0.21	0.97
	Incidental Memory	196	-0.02	-0.23, 0.18	0.82
Gs	Visual matching, WJ-III	195	0.37	0.17, 0.56	<0.001
	Decision speed, WJ-III	195	0.20	0.01, 0.40	0.05

SD: standard error; Gsm: short-term memory; Gc: comprehension and knowledge; Gps: psychomotor speed; Gv: visual processing; Glr: long-term storage and retrieval; Gs: cognitive processing speed; NES2: Neurobehavioral Evaluation System 2;WISC-R: Wechsler Intelligence Scale for Children revised; WAIS-R: Wechsler Adult Intelligence Scale revised; the WMS-III: Wechsler Memory Scale third edition; WJ-III: Woodcock-Johnson III test of cognitive abilities; BNT: Boston Naming Test; CVLT: California Verbal Learning Test (CVLT)

[a] Change in the standard deviation of the neurocognitive test score associated with a 1-SD increase in VO_{2Max}, all analyses were adjusted for sex.

Table S3. Adjusted associations between VO_{2max} and neurocognitive functions accounting for inverse probability weighting

Neurocognitive domain	B (95% CI)[a]	*p*-value
Short term memory	0.21 (-0.04, 0.45)	0.10
Verbal comprehension and knowledge	0.17 (-0.06, 0.40)	0.15
Psychomotor speed	0.03 (-0.18, 0.25)	0.76
Visual processing	0.15 (-0.11, 0.41)	0.27
Long-term storage and retrieval	0.13 (-0.09, 0.34)	0.24
Cognitive processing speed	0.24 (0.01, 0.47)**	0.04
Cognitive efficiency	0.28 (0.02, 0.55)**	0.03
General thinking abilities	0.21 (-0.02, 0.44)*	0.07
General function (g)	0.24 (0.01, 0.47)**	0.04

Models were adjusted for sex, physical activity, smoking status, BMI, family background, and prenatal methylmercury exposure

[a] Change in the standard deviation of the neurocognitive function associated with a 1-SD increase in VO_{2Max}
* <0.10; ** <0.05

Table S4. Adjusted associations between VO_{2max} and neurocognitive functions in regard to prenatal mercury exposure split at the median (23.5 µg/L), accounting for inverse probability weighting

Neurocognitive domain	B (95% CI)[a]		*p*-difference
	Low prenatal exposure (<23.5 µg/L)	High prenatal exposure (≥23.5 µg/L)	
Short term memory	0.14 (-0.19, 0.46)	0.20 (-0.19, 0.58)	0.82
Verbal comprehension and knowledge	0.15 (-0.11, 0.48)	0.14 (-0.22, 0.49)	0.84
Psychomotor speed	0.18 (-0.10, 0.46)	-0.08 (-0.43, 0.26)	0.25
Visual processing	0.17 (-0.14, 0.49)	-0.02 (-0.45, 0.41)	0.48
Long-term storage and retrieval	0.15 (-0.13, 0.43)	0.14 (-0.19, 0.47)	0.96
Cognitive processing speed	0.38 (0.09, 0.67)**	0.06 (-0.30, 0.43)	0.18
Cognitive efficiency	0.34 (0.00, 0.68)**	0.19 (-0.22, 0.60)	0.58
General thinking abilities	0.23 (-0.06, 0.53)	0.16 (-0.20, 0.51)	0.74
General function (g)	0.29 (-0.02, 0.59)*	0.15 (-0.20, 0.49)	0.55

Models were adjusted for sex, physical activity, smoking status, BMI, family background, and prenatal methylmercury exposure

[a] Change in the standard deviation of the neurocognitive function associated with a 1-SD increase in VO_{2Max}

* <0.10; ** <0.05

Table S5. Adjusted associations between VO_{2max} and neurocognitive functions in regard to prenatal mercury exposure split at the 67th percentile (35 µg/L), accounting for inverse probability weighting

Neurocognitive domain	B (95% CI)[a]		*p-difference*
	**Low prenatal exposure (<35 µg/L)**	**High prenatal exposure (≥35 µg/L)**	
Short term memory	0.27 (0.00, 0.57)**	-0.09 (-0.58, 0.40)	0.21
Verbal comprehension and knowledge	0.15 (-0.18, 0.40)	0.16 (-0.25, 0.57)	0.85
Psychomotor speed	0.13 (-0.11, 0.37)	-0.16 (-0.58, 0.27)	0.25
Visual processing	0.29 (0.00, 0.58)*	-0.22 (-0.73, 0.29)	0.08
Long-term storage and retrieval	0.11 (-0.13, 0.35)	0.11 (-0.28, 0.49)	0.99
Cognitive processing speed	0.38 (0.15, 0.60)**	-0.09 (-0.50, 0.32)	0.02
Cognitive efficiency	0.44 (0.16, 0.72)**	-0.16 (-0.68, 0.35)	0.04
General thinking abilities	0.18 (-0.07, 0.44)	0.29 (-0.14, 0.72)	0.67
General function (g)	0.29 (0.03, 0.55)**	0.10 (-0.34, 0.55)	0.47

Models were adjusted for sex, physical activity, smoking status, BMI, family background, and prenatal methylmercury exposure

[a] Change in the standard deviation of the neurocognitive function associated with a 1-SD increase in VO_{2Max}

* <0.10; ** <0.05
